# Homeopathy: What Is It?

**Published:** 1880-06

**Authors:** 


### BIBLIOGRAPHICAL.

HOMEOPATHY: WHAT IS IT?—A statement and Review of its Doctrines and Practice. By A. B. Palmer, A M., M.D., Professor of Pathology and Practice of Medicine in the College of Medicine and Surgery in the University of Michigan, etc. George S. Davis, Medical Publisher, Detroit, 1880.

We have glanced over this effort of the author to present the principles of homeopathy, and find much that coincides with ideas previously entertained. We agree with the author in thinking that the sect should not be persecuted by the regular profession, but the errors embodied in the "koran of their faith" should be charitably dealt with, and their absurdities mildly exposed. We believe that there are many in the faith who are backsliders, and many who are there from purely mercenary motives. Few they are who sincerely and conscientiously adopt Hahnemann's principles, and confine themselves to the practical application of the same.

There is a class who, desiring prosperity with as little work as possible, are easily enticed by the simplicity of some of the homeopathic doctrines: e. g.; that which teaches that the "totality of the symptoms must be the principle—the sole thing the physician has to take note of in *every case* of disease, and to remove by means of his art." Symptoms are classed and grouped together, and remedies are alloted to each with a seeming simplicity charming to one who has no special fondness for study.

The author shows that the principle and only true law of homeopathy—"*similia similitus curantur*"—is fallacious "If there be an identity in the action of the remedy and that of the disease, then can there be no cure but an aggravation? If the similarity or homœpathicity between the action of the remedy, and the disease be too great, then will there be aggravation instead of cure. But in proportion, as the similarity grows less and the difference increases, so will the remedy altar, change, remove, take the place of, or cure the disease. Our remedies then produce an effect different from the disease."

Each doctrine is discussed in detail, and the fallacies set prominently forth, and we cannot discover anything unfair or unjust in the criticisms.

Homœpathy is well enough where "placebo" is the therapeutical indication. Very often it is the case that patients really need no medicine, yet, in their ignorance, are determined to have some. The homœpathic "olfaction" or infinitesimal attenuation can have no toxic affects, and yet satisfies the patient's desire.

Again, routinism blindly exhibits medicine, where, probably, none is required, and where a cure might be perfected by not giving any. In this instance homœpathy would be far preferable to the interference of the *ignorant routinist.*

Let homœpathy run its course and decide its own limitation. The effort of the regular profession should be not to combat homœpathy, but to elevate its own standard of education and render the homœpathist an unnecessary evil.

